# Determination of *N*-Acetyl-L-cysteine Ethyl Ester (NACET) by Sequential Injection Analysis

**DOI:** 10.3390/s24020312

**Published:** 2024-01-05

**Authors:** Lea Kukoc-Modun, Tomislav Kraljevic, Dimitrios Tsikas, Tony G. Spassov, Spas D. Kolev

**Affiliations:** 1 Department of Analytical Chemistry, Faculty of Chemistry and Technology, University of Split, Ruđera Boškovića 35, 21000 Split, Croatia; 2 Department of Chemistry, Faculty of Science and Education, University of Mostar, Matice hrvatske bb, 88000 Mostar, Bosnia and Herzegovina; tomislav.kraljevic325@gmail.com; 3 Institute of Toxicology, Core Unit Proteomics, Hannover Medical School, Carl-Neuberg-Str. 1, 30625 Hannover, Germany; tsikas.dimitros@mh-hannover.de; 4 Faculty of Chemistry and Pharmacy, Sofia University “St. Kl. Ohridski”, 1 James Bourchier Blvd., 1164 Sofia, Bulgaria; tspassov@chem.uni-sofia.bg; 5 School of Chemistry, The University of Melbourne, Melbourne, VIC 3010, Australia; 6 Department of Chemical Engineering, The University of Melbourne, Melbourne, VIC 3010, Australia

**Keywords:** sequential injection analysis (SIA), *N*-acetyl-L-cysteine ethyl ester (NACET), neocuproine (NCN), bicinchoninic acid (BCA), bathocuproine disulfonic acid (BCS)

## Abstract

New sequential injection analysis (SIA) methods with optical sensing for the determination of *N*-acetyl-L-cysteine ethyl ester (NACET) have been developed and optimized. NACET is a potential drug and antioxidant with advantageous pharmacokinetics. The methods involve the reduction of Cu(II) in its complexes with neocuproine (NCN), bicinchoninic acid (BCA), and bathocuproine disulfonic acid (BCS) to the corresponding chromophoric Cu(I) complexes by the analyte. The absorbance of the Cu(I) complexes with NCN, BCA, and BCS was measured at their maximum absorbance wavelengths of 458, 562, and 483 nm, respectively. The sensing manifold parameters and experimental conditions were optimized for each of the Cu(II) complexes used. Under optimal conditions, the corresponding linear calibration ranges, limits of detection, and sampling rates were 8.0 × 10^−6^–2.0 × 10^−4^ mol L^−1^, 5.5 × 10^−6^ mol L^−1^, and 60 h^−1^ for NCN; 6.0 × 10^−6^–1.0 × 10^−4^ mol L^−1^, 5.2 × 10^−6^ mol L^−1^, and 60 h^−1^ for BCA; and 4.0 × 10^−6^–1.0 × 10^−4^ mol L^−1^, 2.6 × 10^−6^ mol L^−1^, and 78 h^−1^ for BCS. The Cu(II)-BCS complex was found to be best performing in terms of sensitivity and sampling rate. Usual excipients in pharmaceutical preparations did not interfere with NACET analysis.

## 1. Introduction

**Figure 1:**
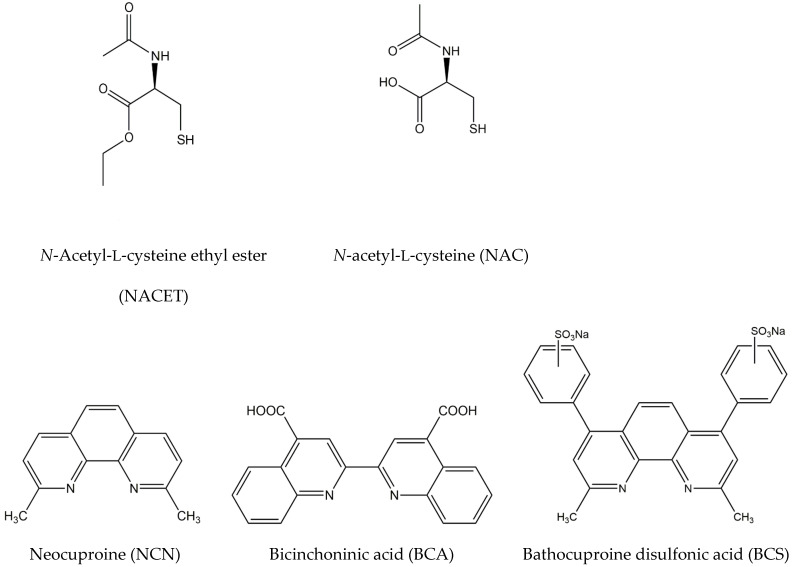
Structural formulas of *N*-acetyl-L-cysteine ethyl ester (NACET), *N*-acetyl-L-cysteine (NAC), neocuproine (NCN), bicinchoninic acid (BCA), and bathocuproine disulfonic acid (BCS).

*N*-acetyl-L-cysteine ethyl ester (NACET, Figure 1) is a lipophilic and charge-free congener of *N*-acetyl-L-cysteine (NAC, Figure 1) with improved pharmacological properties, e.g., antioxidant properties in reducing oxidative stress. The esterification of the carboxyl group of NAC increases its lipophilicity, thus improving its pharmacokinetics. NACET has the potential to substitute NAC as a mucolytic agent but also as an antioxidant, a source of glutathione (GSH), and a paracetamol antidote [1,2].

It has been reported recently that NAC does not act as a GSH enhancer in human endothelial cells in a dose-dependent manner; furthermore, treatment with NACET has an evident ceiling effect [3]. The levels reached by intracellular GSH after treatments with NACET resulted in a balance between the induction of its synthesis (by increased cysteine) and enzyme inhibition (by increased intracellular NAC) [3]. These findings show that NACET is a promising potential drug, and there is a clear need for fast and reliable online methods for its determination in pharmaceutical formulations as part of the quality control step of the corresponding manufacturing processes. However, there are only a few published methods for its determination. These methods include high-performance liquid chromatography (HPLC) [4], kinetic spectrophotometry [5], and flow injection analysis (FIA) with spectrophotometric detection [6], with only the last method allowing online NACET determination. Both spectrophotometric methods mentioned above are based on the reduction of Cu(II) complexes with neocuproine (NCN), bicinchoninic acid (BCA), or bathocuproine disulfonic acid (BCS) (Figure 1) to the corresponding Cu(I)-ligand complexes.

These redox reactions (Equations (1)–(3)) are fast, proceed in a broad pH range, and provide stable signals [5] and, therefore, are suitable for the online spectrophotometric determination of NACET:

2RSH + 2[Cu(NCN)_2_]^2+^ ⇄ RSSR + 2[Cu(NCN)_2_]^+^ + 2H^+^ (1)

2RSH + 2[Cu(BCA)_2_]_2_^2−^ ⇄ RSSR + 2[Cu(BCA)_2_]^3−^ + 2H^+^ (2)

2RSH + 2[Cu(BCS)_2_]_2_^2−^ ⇄ RSSR + 2[Cu(BCS)_2_]^3−^ + 2H^+^ (3)

where RSH represents NACET (a thiol compound).

Flow injection analysis (FIA) procedures allow automatic chemical analysis and significantly reduce sample volumes compared to batch methods. They have been used extensively in pharmaceutical analysis [7,8,9,10,11,12]. However, these procedures usually require relatively large volumes of reagents, which continuously flow through the corresponding manifolds, thus leading to waste generation and high cost of analysis, especially when expensive reagents are used. To overcome these limitations of FIA, Ruzicka and Marshall introduced sequential injection analysis (SIA) in 1990 as an alternative flow-through sample-handling and sensing technique which is economical with respect to both sample and reagent consumption in addition to allowing computer control of most manifold and operational parameters [13]. This flow technique has been successfully applied to the analysis of analytes of pharmaceutical interest [14,15,16,17]. Since the reagents for the spectrophotometric determination of NACET mentioned above (i.e., NCN, BCA, and BCS) are expensive (e.g., €146 per gram of BCS, Sigma-Aldrich, Burlington, MA, USA), SIA is expected to reduce significantly the cost of analysis compared to FIA. Therefore, this study aims to develop SIA methods with optical sensing for the determination of NACET based on the use of the color redox reactions involving the Cu(II) complexes with NCN, BCA, and BCS (Equations (1)–(3)) which will be applicable to the online determination of NACET as part of the quality control step of the manufacturing of its future pharmaceutical preparations. Since such pharmaceutical preparations are still not in production, the utility of the newly developed SIA methods was assessed on the basis of interferences of common excipients that might be present in future NACET pharmaceutical formulations.

## 2. Materials and Methods

### 2.1. Solution Preparation

All chemicals were of analytical-reagent grade, and all the solutions were made in Milli-Q (Millipore, Burlington, VT, USA, resistance > 18 MΩ) deionized water.

The NACET (C_7_H_13_NO_3_S, MW 191.2, mp 44.1–44.5 °C) synthetic procedure, compound purification, and characterization by mass and infrared spectrometry, ^1^HNMR, and polarimetry were reported by Tsikas et al. [18].

A 1.0 × 10^−2^ mol L^−1^ stock solution of NACET was prepared by dissolving 191.2 mg of NACET in a 100 mL Britton–Robinson buffer solution (pH = 2). The stock solution was found to be stable for one month when kept at 4 °C in a refrigerator. Working standard solutions were freshly prepared by appropriate dilution of the stock solution with different buffer solutions (Britton–Robinson buffer, phosphate buffer, and acetate buffer), the preparation of which is described elsewhere [5]. The desired pH of each working solution was adjusted by adding 2.0 mol L^−1^ sodium hydroxide solution.

A stock solution of 1.0 × 10^−2^ mol L^−1^ of Cu(II) was prepared by dissolving 0.1248 g of CuSO_4_.5H_2_O in 50.0 mL deionized water.

Cu(II)-NCN reagent was prepared by dissolving 25.0 mg of CuSO_4_.5H_2_O and 50.0 mg of NCN in 250 mL of Britton–Robinson buffer solution (pH = 5).

A stock solution of BCA (4.0 × 10^−3^ mol L^−1^) was prepared by dissolving 77.6 mg of BCA disodium salt in 50 mL phosphate buffer solution (pH = 7). This solution (20 mL) was mixed with 4 mL of 1.0 × 10^−2^ mol L^−1^ Cu(II) stock solution and diluted with phosphate buffer solution (pH 7) to 100 mL to obtain the Cu(II)-BCA reagent solution containing 4.0 × 10^−4^ mol L^−1^ Cu(II) and 8.0 × 10^−4^ mol L^−1^ BCA.

A BCS stock solution (2.0 × 10^−3^ mol L^−1^) was prepared by dissolving 58.2 mg of BCS disodium salt in 50.0 mL of deionized water. The Cu(II)-BCS reagent solution (100 mL) containing 4.0 × 10^−4^ mol L^−1^ Cu(II) and 8.0 × 10^−4^ mol L^−1^ BCS was prepared by mixing 4 mL of 1.0 × 10^−2^ mol L^−1^ Cu(II) stock solution, 40 mL 2.0 × 10^−3^ mol L^−1^ BCS stock solution, and 56 mL of acetate buffer solution (pH = 5).

Solutions for the interference studies (0.2 mol L^−1^) which contained common ions (K^+^, Na^+^, NO_3_^−^ and SO_4_^2−^) and excipients in pharmaceutical preparations were prepared in deionized water by dissolving KNO_3_, Na_2_SO_4_, D-(+)-glucose, D-(−)-fructose, lactose, citric acid, L-(+)-tartaric acid, and boric acid, all purchased from Kemika (Zagreb, Croatia). The appropriate volumes of these solutions were added to appropriately buffered 4.0 × 10^−5^ mol L^−1^ NACET solutions to form final molar ratios of NACET: interferent of 1:5, 1:10, 1:50, 1:100, 1:250, and 1:500.

### 2.2. Apparatus

**Figure 2:**
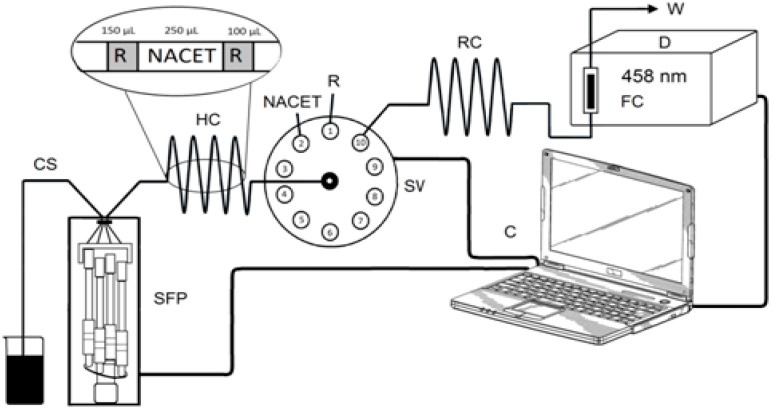
Schematic diagram of the SIA manifold: SFP–syringe-free pump (M50), SV—10-port selection valve, HC–holding coil, RC—reaction coil, CS—carrier stream (deionized H_2_O), R—reagent, NACET (*N*-acetyl-L-cysteine ethyl ester) solution, FC—flow cell, W—waste, C—computer. Inset: optimal aspiration sequence.

The SIA manifold (Figure 2) incorporated a syringe-free stepper-motor-driven pump (Cheminert^®^ M50) linked to a programmable microstepping driver (Micro Lynx 4, Intelligent Motion Systems, Marlborough, MA, USA) and a 10-port selection valve (C25-3180EMH) with a multi-position actuator control module (EMHCA-CE), all purchased from VICI Valco Instruments (Houston, TX, USA) and controlled using M6-LHS software (Version 1.0.5e, VICI Valco Instruments). The absorbance of the Cu(I) complexes with NCN, BCA, and BCS was measured at 458, 562, and 483 nm, respectively, using a double-beam UV/Vis spectrophotometer equipped with a 1 cm flow-through cell of 80 μL volume (UV-1601, Shimadzu, Kyoto, Japan). The output signals with a data acquisition frequency of 1 Hz were recorded by interfacing the spectrophotometer with a computer running UVProbe software (Version 2.31, Shimadzu). The holding coil (HC, 120.0 cm) and the reaction coil (RC, 30.0 cm), as well as the rest of the manifold tubing (0.8 mm id), were made of polytetrafluoroethylene.

### 2.3. Analytical Procedure

Prior to each measurement, the holding and reaction coils were filled with carrier solution (deionized H_2_O) by selecting port 10 of the selection valve. A stable baseline was achieved by dispensing 3000 μL of carrier solution.

The optimized analytical steps for the determination of NACET with the newly developed SIA manifold are summarized in Table 1. A “sandwich” aspiration sequence was used, which involved aspiration of the sample/standard between two zones of the reagent. This configuration resembled the flow configuration in a FIA manifold developed previously by us [7].

The syringe-free pump sequentially loaded 150 µL of reagent, 250 µL of sample/standard, and an additional 100 µL of reagent into the holding coil (HC) at a flow rate of 3000 µL min^−1^, where they merged into a reaction zone.

By reversing the flow, the reaction zone was propelled through the reaction coil (RC) towards the spectrophotometric flow cell (FC) at the same flow rate of 3000 µL min^−1^. The passage of the colored product through the FC resulted in a transient absorbance peak, the height of which was the analytical signal, which was proportional to the concentration of NACET in the sample/standard. All measurements were performed in triplicate.

### 2.4. Optimization of the Design and Operational Parameters

The most important design and operational parameters were optimized using a univariate approach, and the corresponding initial values, optimal values, and studied ranges are summarized in Table 2, where the parameters optimized are listed in the order in which optimization was carried out. The NACET concentration in the optimization experiments was 4.0 × 10^−5^ mol L^−1^. The NACET concentration range of 1.0 × 10^−5^–1.0 × 10^−4^ mol L^−1^ corresponded to an absorbance range of up to 0.8 a.u. and, therefore, this range was considered to be optimal for reliable analysis.

## 3. Results and Discussion

In the color reactions used in this study (Equations (1)–(3)), NACET reduced the NCN, BCA, and BCS complexes with Cu(II) to the corresponding Cu(I) colored complexes with absorption maxima at 458 nm, 562, nm and 483 nm, respectively [6]. The optimal pH conditions for conducting these redox reactions were determined in a previous study [5] as 5.0, 7.0, and 5.0, respectively. Therefore, all three Cu(II)-ligand reagents were adjusted to their corresponding optimal pH values.

### 3.1. Method Optimization

The optimization of all parameters was conducted with respect to the analytical signal (*A*), which was the absorbance peak maximum.

#### 3.1.1. Flow Rate

**Figure 3:**
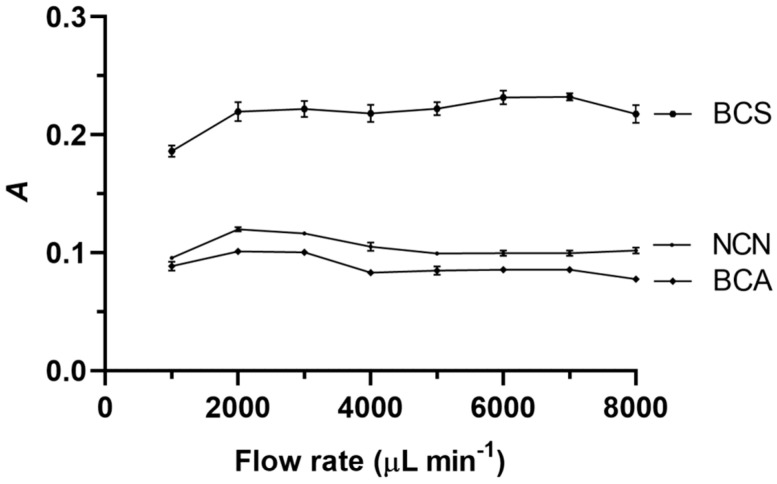
Effect of the carrier stream flow rate on the analytical signal (*A*) when NCN, BCA, and BCS were used. Experimental conditions: aspiration sequence—reagent–standard; reagent volume—250 μL; sample (standard) volume—250 μL; holding coil volume—1000 μL; reaction coil length—70 cm; reagent concentration—4.0 × 10^−4^ mol L^−1^; and NACET concentration—4.0 × 10^−5^ mol L^−1^. Error bars are ± standard deviation (SD).

The carrier stream flow rate for both aspiration of the standards and reagents and the propelling of the corresponding zones from the holding coil to the detector were maintained the same, and their effect on the analytical signal is illustrated in Figure 3. The flow rate effect for all three reagents was not significant. This can be explained by the mass transfer and kinetic effects, which influence the flow rate effect differently. The analytical signal should increase with the flow rate in the absence of a chemical reaction. However, if a chemical reaction is involved, an increase in the flow rate will reduce the amount of detectable product generated. Therefore, the results presented in Figure 3 suggest that these two processes balanced each other. The optimal flow rate values in the cases of both NCN and BCA were found to be the same, i.e., 3000 μL min^−1^, while the optimal value when BCS was used was determined as 6000 μL min^−1^.

#### 3.1.2. Aspiration Sequence and Reagent Volume

**Figure 4:**
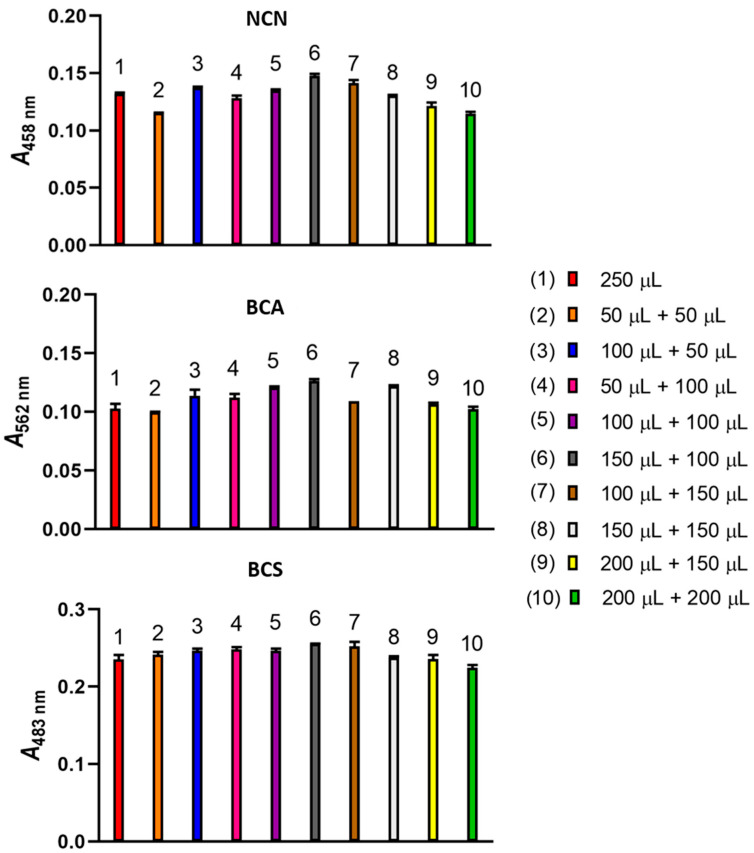
Effect of the Cu(II)-NCN, Cu(II)-BCA, and Cu(II)-BCS reagent volumes and sequence of aspiration on the analytical signal. Experimental conditions, as in Figure 3, except for carrier flow rates of 3000, 3000, and 6000 μL min^−1^ when NCN, BCA, and BCS were used, respectively. Error bars are ±SD.

Initially, two different aspiration sequences of a NACET standard and Cu(II)-NCN reagent solutions, both 250 μL, were compared, i.e., 250 μL Cu(II)-NCN reagent solution followed by 250 μL NACET standard solution and 250 μL NACET standard solution followed by 250 μL Cu(II)-NCN reagent solution. The latter sequence produced a 10% increase in the analytical signal. Then, the analytical signals produced by a number of aspiration sequences involving sandwiching the NACET standard zone between two Cu(II)-NCN reagent zones were compared (Figure 4). The highest analytical signal was obtained in the aspiration sequence 150 μL Cu(II)-NCN reagent solution—250 μL NACET standard solution—100 μL Cu(II)-NCN reagent solution. Similar results were obtained when the Cu(II)-BCA and Cu(II)-BCS reagents were used (Figure 4). Therefore, this aspiration sequence was used in the subsequent experiments for all three reagents.

#### 3.1.3. Sample Volume and Holding Coil Length

**Figure 5:**
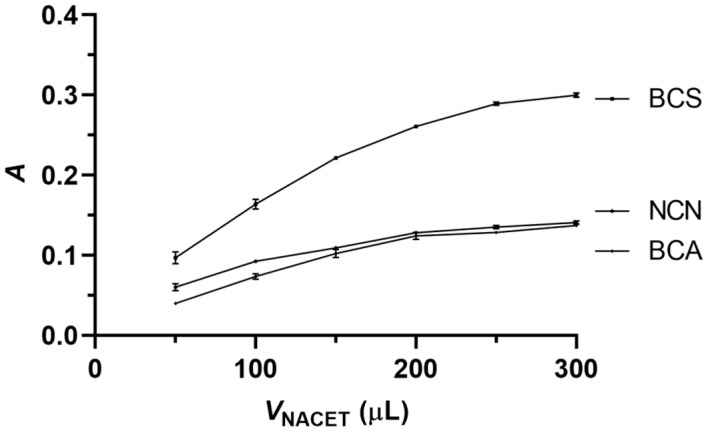
Influence of the volume of the NACET standard on the analytical signal. Experimental conditions, as in Figure 4, except for the reagent volume of 250 μL and the aspiration sequence of 150 μL reagent solution—NACET standard solution—100 μL reagent solution. Error bars are ±SD.

Figure 5 shows the effect of the NACET standard volume on the analytical signal. In the cases of all three reagents, the analytical signal increased initially with the volume of the NACET standard and then leveled off at 250 μL, which value was selected as the optimal sample/standard volume for all three reagents.

The effect of holding coils (Figure 2) of two different volumes (500 and 1000 μL) on the analytical signal was studied with all three reagents. There was no statistically significant difference in the results for these two volumes, and therefore, the shorter holding coil (i.e., 500 μL) was used in the subsequent experiments for all three reagents.

#### 3.1.4. Reaction Coil

**Figure 6:**
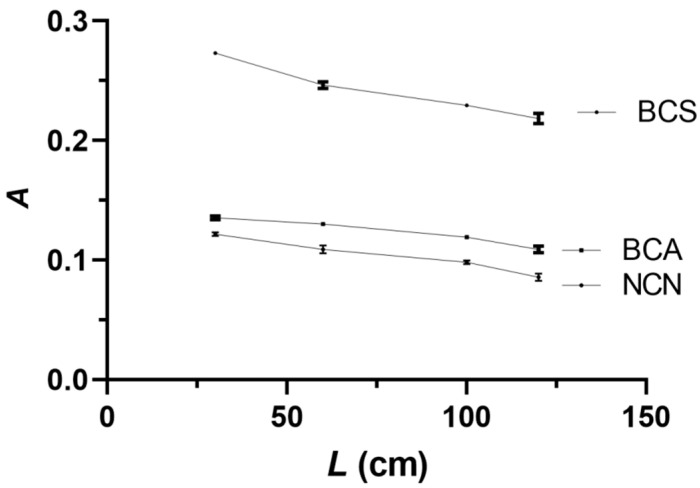
Effect of the reaction coil length (*L*) on the analytical signal. Experimental conditions as in Figure 5, except for NACET sample/standard volume of 250 μL. Error bars are ±SD.

The influence of the reaction coil length (*L*) on the analytical signal was investigated in the range of 30–150 cm. Shorter than 30 cm reaction coils could not be used because of spatial constraints. The results, shown in Figure 6, indicate that for all three reagents, the highest analytical signal values were obtained with the shortest possible reaction coil of 30 cm, and this reaction coil value was selected as optimal.

#### 3.1.5. Reagent Concentration

Calibration curves for the NACET were obtained by the method of Miller and Miller [19] for all three reagents by varying their concentrations in the range 1.0 × 10^−4^–1.0 × 10^−3^ mol L^−1^ in the cases of Cu(II)-NCN and Cu(II)-BCS. Due to its low water solubility, the effect of the Cu(II)-BCA reagent was studied only in the range 1.0 × 10^−4^–4.0 × 10^−4^ mol L^−1^. It was observed that for NACET concentrations lower than 8.0 × 10^−6^ mol L^−1^ in the case of Cu(II)-NCN, 6.0 × 10^−6^ mol L^−1^ in the case of Cu(II)-BCA and 4.0 × 10^−6^ mol L^−1^ in the case of Cu(II)-BCS the absorbance peaks were distorted (i.e., mostly double-humped peaks were obtained) and therefore results for lower NACET concentrations were not used in the calibration process. In all three cases, the calibration curve slope initially increased with the reagent concentration and started leveling off at 4.0 × 10^−4^ mol L^−1^, and this value was selected as the optimal reagent concentration for all three reagents.

### 3.2. Analytical Figures of Merit

**Figure 7:**
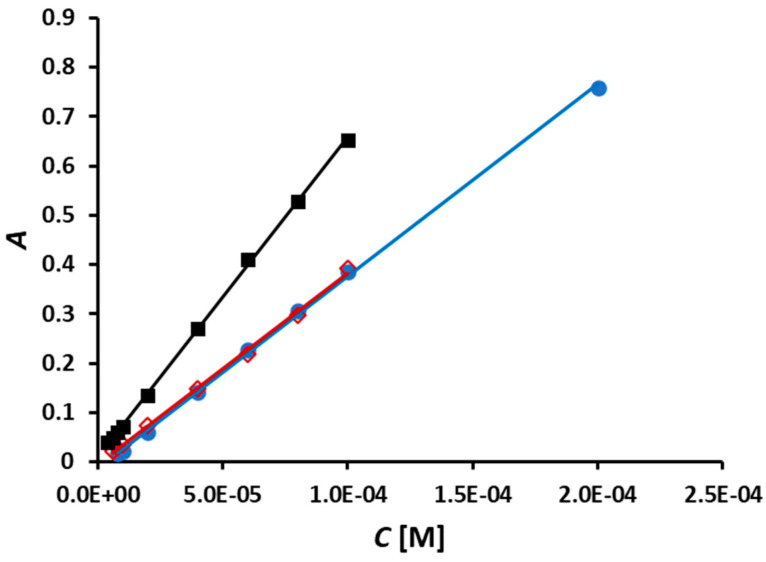
Calibration curves for the SIA determination of NACET obtained under optimal conditions (Table 2) with reagents Cu(II)-NCN (●), Cu(II)-BCA (**◇**), and Cu(II)-BCS (■).

The methods involving the use of NCN-, BCA-, and BCS-based Cu(II) reagents were calibrated under optimal conditions (Figure 7).

The analytical figures of merit of these methods are listed in Table 3. As mentioned above, very low NACET concentrations and calibration blanks produced distorted peaks in the cases of all three reagents and, therefore, were not used in constructing the corresponding three calibration curves. For this reason, the limit of detection (LOD) values were calculated by the method of Miller and Miller [19], based on regression statistics, instead of using the conventional method based on the standard deviation of calibration blanks or the baseline. The slopes of the three calibration curves (Figure 7, Table 3) are consistent with the molar absorptivity values of the corresponding Cu(I) complexes (i.e., 7.9 × 10^3^ L mol^−1^ cm^−1^ for Cu(I)-NCN [20], 7.7 × 10^3^ L mol^−1^ cm^−1^ for Cu(I)-BCA [21], and 1.3 × 10^4^ L mol^−1^ cm^−1^ for Cu(I)-BCS [20]). The sampling rate at the optimal carrier stream flow rate (Table 2) was found to be 60 h^−1^ in the cases of the Cu(II)-NCN and Cu(II)-BCA reagents and 78 h^−1^ in the case of the Cu(II)-BCS reagent.

The LOD values calculated by the method of Miller and Miller [19] are generally higher than values calculated on the basis of the standard deviation of calibration blanks or the baseline. Therefore, while the RSD and sampling rate values of the newly developed SIA method were similar to those of the only other NACET method online (i.e., the FIA method [6]), the LOD values were approximately an order of magnitude higher. However, when the FIA data were processed by the method of Miller and Miller, the LOD values (i.e., 3.4 × 10^−6^, 2.9 × 10^−6^, and 2.9 × 10^−6^ mol L^−1^ for the methods based on the use of Cu(II)-NCN, Cu(II)-BCA, and Cu(II)-BCS, respectively) were found to be very similar to those of the SIA methods (Table 3). It should also be pointed out that the newly developed SIA method is expected to be used as a quality control tool in the manufacturing of NACET-based pharmaceutical preparations where very high sensitivity will not be required. An important advantage with respect to the SIA method is the fact that NACET analysis is approximately three times cheaper than analysis conducted by its FIA counterpart.

### 3.3. Interferences

The interference effect of common inorganic ions, included in KNO_3_ and Na_2_SO_4_, and common excipients, which can potentially be included in the pharmaceutical preparations incorporating NACET, was studied. The interferents were present in molar excesses of 5, 10, 50, 100, 250, and 500. Table 4 includes the highest NACET-to-interferent molar ratios where the absolute error in the determination of NACET was lower than 5%.

The results in Table 4 show that sugar excipients did not interfere up to 500 times molar excess. Similar results were obtained for boric acid, KNO_3_, and Na_2_SO_4_. As expected, interferences, consistent with the stability of the Cu(II) complexes with NCN (log*β*_2_ = 11.7 [22]), BCA (log*β*_2_ = 8.9 [23]) and BCS (log*β*_2_ = 12.4 [24] where *β*_2_ is the overall stability constant) (Equations (1)–(3)) were observed with compounds capable of forming complexes with Cu(II), i.e., citric and tartaric acids. However, it should be pointed out that these interferences were negligible in the case of BCS.

## 4. Conclusions

Three SIA methods for the online determination of NACET utilizing the color reagents Cu(II)-NCN, Cu(II)-BCA, or Cu(II)-BCS were developed, optimized, and studied for possible interferences by common excipients. The LODs for these reagents were determined as 5.5 × 10^−6^, 5.2 × 10^−6^, and 2.6 × 10^−6^ mol L^−1^, respectively, while the corresponding sampling rates were 60, 60, and 78 h^−1^. On the basis of the results obtained, it was concluded that the method using the Cu(II)-BCS reagent provided the highest sensitivity and sampling rate. In addition, this method was practically immune to interferences by common excipients, expected to be present in future NACET pharmaceutical preparations. In comparison with the only other flow analysis method for NACET, which is based on flow injection analysis principles, the newly developed SIA method is significantly more economical with respect to reagent consumption, thus reducing the cost of analysis without compromising on sensitivity. It can be expected that the newly developed SIA methods, especially the one based on the use of the Cu(II)-BCS reagent, will play an important role in the quality control step of the manufacturing of NACET pharmaceutical preparations in the future.

## Figures and Tables

**Table 1:** Optimized SIA procedure.

Step	Valve Position	Operation Time (s) *	Flow Rate (μL min^−1^)	Volume (μL)	Operation Description
1	1	3	3000	150	Aspirating reagent to the holding coil
2	2–9	5	3000	250	Aspirating sample to the holding coil
3	1	2	3000	100	Aspirating reagent to the holding coil
4_NCN, BCA_	10	60	3000	3000	Propelling of the reaction mixture to the flow cell
4_BCS_	10	30	6000	3000	

* Note: Volume and flow rate were assigned while the operation time was calculated.

**Table 2:** Initial values, studied ranges, and optimal values of the main design and operational parameters.

Parameter	Initial Values	Studied Range	Optimal Value		
			NCN Method	BCA Method	BCS Method
Carrier stream flow rate (μL min^−1^)		1000–10,000	3000	3000	6000
Aspiration sequence	Reagent–standard	(1) Reagent–standard (2) Standard–reagent (3) Reagent–standard–reagent	3	3	3
Reagent volume (μL)	250	50–450	250 (150 + 100)	250 (150 + 100)	250 (150 + 100)
Sample volume (μL)	250	50–450	250	250	250
Holding coil volume (μL)	1000	500, 1000	500	500	500
Reaction coil length (cm)	70	30–120	30	30	30
Reagent concentration (mol L^−1^)	4.0 × 10^−4^	1.0 × 10^−4^–1.0 × 10^−3^	4.0 × 10^−4^	4.0 × 10^−4^	4.0 × 10^−4^

**Table 3:** Analytical figures of merit.

	Cu(II)-NCN	Cu(II)-BCA	Cu(II)-BCS
Linear range (mol L^−1^)	8 × 10^−6^–2 × 10^−4^	6 × 10^−6^ –1 × 10^−4^	4 × 10^−6^–1 × 10^−4^
Calibration equation (A = aC + b)			
a ± SD	(3.893 ± 0.041) × 10^3^	(3.863 ± 0.070) × 10^3^	(6.477 ± 0.055) × 10^3^
b ± SD	(1.247 ± 0.369) × 10^−2^	−(4.696 ± 3.689) × 10^−2^	(9.562 ± 2.737) × 10^−3^
LOD (mol L^−1^)	5.5 × 10^-6^	5.2 × 10^−6^	2.6 × 10^−6^
RSD at 8 × 10^−6^ and 4 × 10^−5^ mol L^−1^ (%)	1.6/1.7	1.2/2.5	0.7/0.9
Sampling rate (h^−1^)	60	60	78

**Table 4:** Interference effects in the methods for the determination of NACET using NCN, BCA, and BCS.

Interferent	NACET *: Interferent (Molar Ratio)		
	NCN	BCA	BCS
Glucose	1:500	1:500	1:500
Fructose	1:500	1:500	1:500
Lactose	1:500	1:500	1:500
Citric acid	1:10	1:5	1:250
Tartaric acid	1:10	1:5	1:500
Boric acid	1:500	1:500	1:500
Na_2_SO_4_	1:500	1:500	1:500
KNO_3_	1:500	1:500	1:500

* [NACET] = 4 × 10^−5^ mol L^−1^.

## Data Availability

Data will be provided upon request.
